# Can self-powered piezoelectric materials be used to treat disc degeneration by means of electrical stimulation?

**DOI:** 10.3389/fbioe.2024.1397261

**Published:** 2024-05-09

**Authors:** Huagui Huang, Kaizhong Wang, Xianyan Liu, Jinzuo Wang, Moran Suo, Xin Liu, Zhonghai Li

**Affiliations:** ^1^ Department of Orthopedics, First Affiliated Hospital of Dalian Medical University, Dalian, China; ^2^ Key Laboratory of Molecular Mechanism for Repair and Remodeling of Orthopedic Diseases, Dalian, China

**Keywords:** intervertebral disc, intervertebral disc degeneration, mechanotransduction, piezoelectricity, piezoelectric materials

## Abstract

Intervertebral disc degeneration (IDD) due to multiple causes is one of the major causes of low back pain (LBP). A variety of traditional treatments and biologic therapies are currently used to delay or even reverse IDD; however, these treatments still have some limitations. Finding safer and more effective treatments is urgent for LBP patients. With increasing reports it has been found that the intervertebral disc (IVD) can convert pressure loads from the spine into electrical stimulation in a variety of ways, and that this electrical stimulation is of great importance in modulating cell behavior, the immune microenvironment and promoting tissue repair. However, when intervertebral disc degeneration occurs, the normal structures within the IVD are destroyed. This eventually leads to a weakening or loss of self-powered. Currently various piezoelectric materials with unique crystal structures can mimic the piezoelectric effect of normal tissues. Based on this, tissue-engineered scaffolds prepared using piezoelectric materials have been widely used for regenerative repair of various types of tissues, however, there are no reports of their use for the treatment of IDD. For this reason, we propose to utilize tissue-engineered scaffolds prepared from piezoelectric biomaterials with excellent biocompatibility and self-powered properties to be implanted into degenerated IVD to help restore cell type and number, restore extracellular matrix, and modulate immune responses. It provides a feasible and novel therapeutic approach for the clinical treatment of IDD.

## 1 Introduction

Low back pain (LBP) is becoming increasingly common in today’s society as the global population grows and ages. Globally, life expectancy with disability due to low back pain increased by 54% between 1990 and 2015, making it the leading cause of disability worldwide (Hartvigsen et al., 2018). The etiology and pathology of LBP is relatively complex, and a specific injurious cause cannot be identified for almost all patients with low back pain. Instead, intervertebral disc degeneration (IDD) is considered one of the leading causes of LBP (Golob and Wipf, 2014). The etiology and pathology of low back pain is relatively complex, and almost all low back pain sufferers are unable to identify a specific cause of injury. IDD is considered one of the leading causes of low back pain. The normal intervertebral disc (IVD), located between the vertebrae, ligaments and surrounding muscles, is the most basic motor unit that constitutes the spine. The gelatinous nucleus pulposus (NP), the annulus fibrosus (AF) surrounding the NP, and the cartilaginous endplate (CEP) immediately above and below the upper and lower cones are the main structures that together participate in a variety of physiologic activities. The NP is composed of water, type II collagen, proteoglycans, and various types of cells that remain highly hydrated. The AF is a unique structure consisting of 15–25 concentric rings. The AF surrounds the NP, withstands pressure from all directions, and plays a protective role in the NP. The cartilaginous endplate (CEP), on the other hand, is a thin layer of hyaline cartilage that acts as a medium for transmitting forces in multiple directions between the IVD and the vertebral body. Since the IVD is the largest avascular structure in the body, nutrient transportation is mainly exchanged through the cartilaginous endplate (Li et al., 2021). Early researchers believed that repetitive long-term sustained mechanical loading and wear and tear was the main cause of IDD, but with the latest family and twin studies suggesting that genetic factors play an important role. In addition, cellular senescence, inadequate nutritional supply, trauma, obesity, and smoking have been reported to be high risk factors for the development of IDD (Battié et al., 1991; Kerttula et al., 2000; Oichi et al., 2020). When IDD occurs, the phenotype of cells in the IVD is altered, their number decreases, the expression of enzymes mediating extracellular matrix (ECM) degradation rises, the metabolic processes of synthesis and catabolism of the ECM are imbalanced, and various types of inflammatory cells are infiltrated and activated (Ohnishi et al., 2022). As IDD progresses NP hydration decreases, leading to reduced elasticity. Pressure from the cone in all directions is dispersed to the AF, resulting in altered biomechanical function and structural damage to the AF. Calcification of the CEP further affects the exchange of substances and exacerbates metabolic disturbances within the IVD. Nerve compression by the protruding NP and stimulation by inflammatory factors cause or exacerbate pain and aggravate mobility disorders (Choi et al., 2015).

Currently, it is important to choose the appropriate treatment for patients with different degrees of IDD. Non-surgical treatment is the mainstay of pain management for patients with no symptoms or only mild symptoms that do not interfere with daily life, and 60%–90% of patients can receive non-surgical treatments such as pharmacologic treatments, non-pharmacologic treatments, and interventional treatments (Chiu et al., 2015). However, due to the irreversible nature of IDD, surgical treatments such as spinal decompression, spinal fusion and total disc replacement are still considered as the best options for patients with advanced IDD. Surgical treatments may result in better pain relief, functional improvement, and clinical satisfaction, but long-term follow-up has shown no useful differences in disability outcomes whether surgical or non-surgical treatments are used (Atlas et al., 2001). Based on this, researchers have begun to focus on biological therapies that retard or regenerate disc structure and function. Gene therapy, extracellular vesicle therapy, cell therapy, and tissue engineering therapy show promising applications, on the other hand, potential injection-related risks, ethical and cost issues of biologics simultaneously limit the clinical application of biologic therapies (Hwang et al., 2018). Electrical stimulation therapy, on the other hand, is considered to be one of the good alternative strategies to biological therapy.

In natural bone, external stress-induced remodeling of bone is closely associated with calcium channels mediated by changes in local potentials (Ali et al., 2023). And similarly load-induced potentials are observed within the normal IVD, which mainly include flow and diffusion potentials (Lai et al., 2000). This electrical stimulation promotes the emergence of extensive annular regeneration in the isolated IVD of pigs and upregulates the production of intervertebral disc matrix macromolecules glycans, collagen II and sulphated glycosaminoglycans (sGAGs) through a mechanism associated with BMPs (Kim et al., 2009; Kanan et al., 2022). This piezoelectric effect, which converts mechanical stimuli into electrical stimuli, is widespread in living organisms, ranging from very basic building blocks (amino acids, peptides, proteins, etc.), to highly organized tissues (i.e., bone, skin, etc.) (Yuan et al., 2019). Based on this, researchers have developed a series of piezoelectric materials that can be used for tissue engineering by deforming them to cause asymmetric movement of ions or charges in the material to produce electrical stimulation, with encouraging results. The use of piezoelectric materials as tissue-engineered scaffolds enables electrical *in vivo* electrical stimulation without the need for an external power sources. The use of piezoelectric materials as tissue-engineered scaffolds enables *in vivo* electrical stimulation without the need for an external power source. In addition, such scaffolds should have appropriate structural and mechanical properties to support cell adhesion, proliferation and differentiation. As a major weight-bearing part of the human body, the implanted piezoelectric bone tissue-engineered scaffold can be subjected to multiple stresses within the IVD, thus providing stable electrical stimulation for the purpose of repairing IVD. This self-powered novel piezoelectric tissue-engineered scaffold may be used as a potential modality for the treatment of IDD, which may achieve the goal of solving patients’ low back pain problems in the future.

## 2 Hypothetical

The strain-generated potential (SGP), which converts pressure signals from the spine into electrical signals, has been shown to play an important role in maintaining the normal structure and function of the IVD and regulating cellular behavior. However, with the development of IDD, the cells and various functional components within the IVD are altered, resulting in a weakening of the regulatory role of SGP. Currently, piezoelectric biomaterials with good biocompatibility can well mimic the piezoelectric properties of normal tissues and are now widely used for tissue regenerative repair. Therefore, assuming that piezoelectric tissue engineering scaffolds with self-powered properties are directly implanted into IVD, electrical stimulation can be utilized to achieve regenerative repair of IDD without relying on an external power source.

## 3 Piezoelectric effect of IVD

**FIGURE 1:**
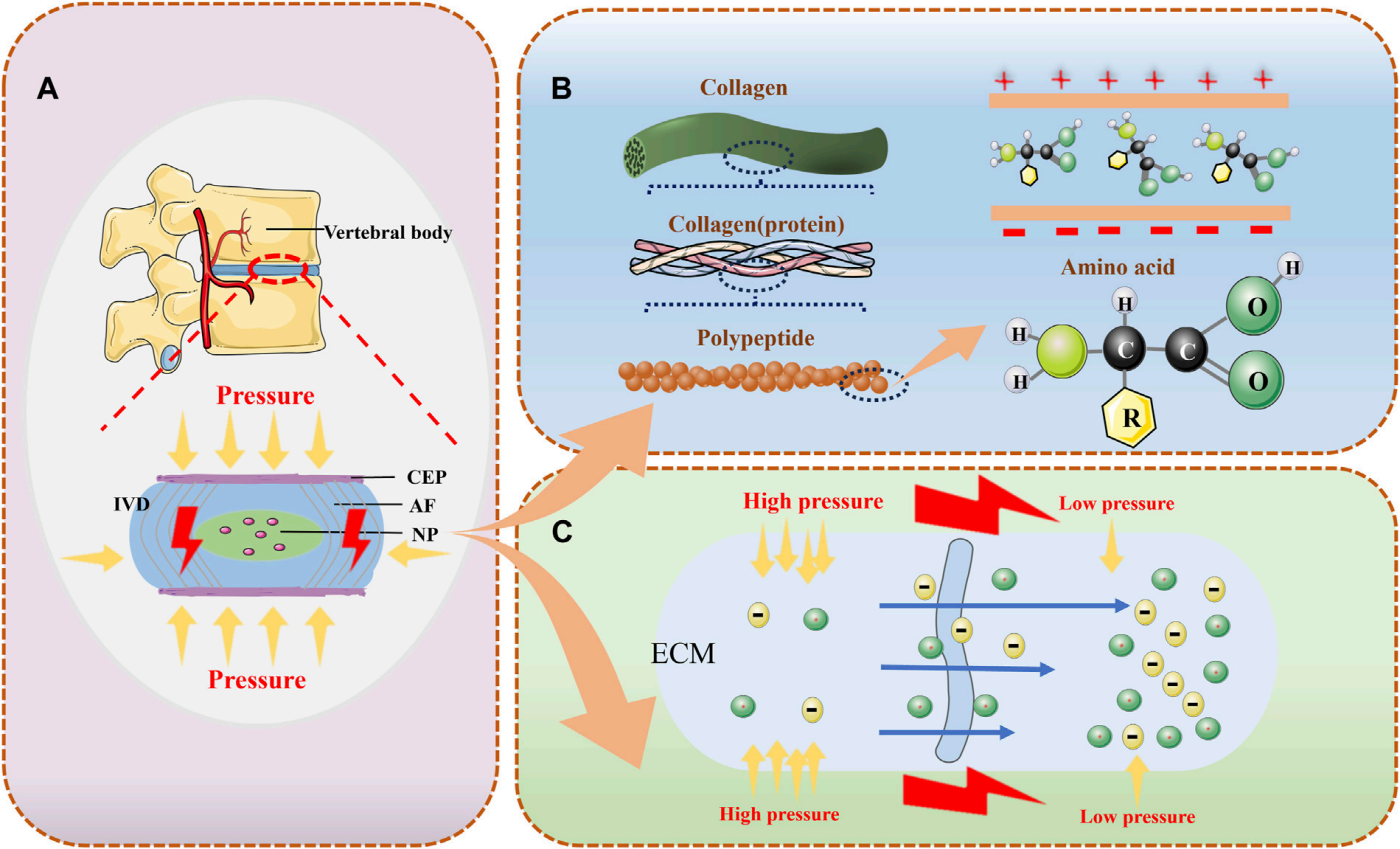
The normal intervertebral disc can provide electrical generation in a variety of ways. **(A)** the IVD can convert pressure signals from the cone into electrical signals; **(B)** the IVD possesses various types of piezoelectric molecules that can provide piezoelectric effects to generate electricity; and **(C)** the various types of ions in the ECM permeate with the solution under different levels of pressure to generate electrical potentials.

In cartilaginous tissues such as articular cartilage and IVD, there is stress load-mediated remodeling of tissue structure. Cells in the ECM sense changes in stress and regulate synthetic and catabolic metabolism through a series of signaling pathways (Fearing et al., 2018). The main reason for this series of changes is due to multiple mechanotransduction pathways that play a huge role in it. However, there is still a lack of sufficient studies to provide a comprehensive and detailed account of the various mechanotransduction pathways involved. However, SGP is now a recognized transduction mechanism in response to pressure loading in tissues (Poillot et al., 2021), and piezoelectric effects and flow potentials are the main reasons for SGP generation. Due to the presence of a variety of non-centrosymmetric molecules within the IVD, loaded pressure loads cause them to deform and thus generate an electrical charge. This phenomenon was first found to exist in bone and plays an important role in tissue regeneration, repair and remodeling (Kim et al., 1995). In addition, the generation of the flow potential is the result of pressure loading that directly helps the charged ions within the IVD to undergo directional flow within the ECM, which passively generates the fluid potential (Figure 1) (Guerin et al., 2018). By dehydrating the IVD tissue the ionic flow can be avoided thus separating the piezoelectric effect of the IVD and the flow potential. The surface of dehydrated IVD after axial pressure loading generated a voltage of ∼1 nV. And compared to the NP, which consists of disorganized collagen fibers passing through a proteoglycan-rich matrix, the AF, which consists of thin sheets of parallel collagen fibers, exhibits a higher piezoelectric effect (Poillot et al., 2020).

Currently, voltage-gated ion channels (VGICs) are considered to be the main modality by which electrical stimulation regulates various physiological functions within the IVD. Electrical stimulation through stress can attract positively charged helices in the voltage sensing domain within the VGICs to the negatively charged side of the membrane. This results in the opening and closing of the channel allowing for the exchange of ions specific to that channel (de Lera Ruiz and Kraus, 2015). Among the many channels, voltage-gated calcium channels are considered to be the most important. As the activity of intracellular calcium concentration increases, calmodulin and calmodulin phosphatase are successively activated. This dephosphorylates NF-AT (nuclear factor of activated cells), which is further translocated into the nucleus where it binds synergistically with other transcription factors to the regulatory regions of inducible genes. These genes further induce the translation of several growth factors, thereby promoting cell proliferation, differentiation and ECM synthesis (More and Kapusetti, 2017). Upon inhibition of L-type calcium channels, the expression of genes such as type Ⅰa collagen, aggrecan and ADAMTS-4 within the IVD undergoes significant downregulation (Poillot et al., 2022). However, with the development of IDD, the fixed charge density and the number of non-centrosymmetric molecules within the IVD are reduced, which can lead to a decrease in the electrical potential within the IVD thereby affecting its structure and function (Yao and Gu, 2007). How to help establish a normal electrophysiological microenvironment in degenerating IVD should be an issue that researchers should consider.

## 4 Piezoelectric biomaterials

The piezoelectric effect plays an important role in cell growth and differentiation, as well as in tissue repair, as the primary means of generating electrical stimulation in various organisms. However, when bone defects and IDD occur, it is difficult for the residual normal tissue to continue to generate sufficient electrical stimulation to aid in tissue repair. Piezoelectric materials can generate an electrical charge on two opposing surfaces in response to mechanical strains, such as stretching, compression, or bending. Therefore, tissue-engineered scaffolds constructed using piezoelectric materials show promising applications in regenerative repair of tissues. This novel tissue-engineered scaffold has self-powered properties and does not require an external source to apply electrical stimulation or implant electrodes. This electrical stimulation promotes tissue regeneration in terms of modulating inflammation, promoting proliferation and aiding remodelling. In the inflammatory phase, electrical stimulation increases blood flow and tissue oxygenation supply to inhibit bacterial growth and minimise wound oedema. In the proliferative phase, electrical stimulation accelerates tissue contraction, fibroblast proliferation, angiogenesis and collagen deposition. In the remodelling phase, electrical stimulation enhances collagen maturation and remodelling, thereby accelerating tissue contraction and increasing tensile strength (Xu et al., 2021). Depending on the source, piezoelectric materials can be broadly classified into piezoelectric ceramics and piezoelectric polymers.

Piezoelectric ceramics such as lead zirconate titanate, barium titanate (BT), zinc oxide, potassium potassium niobate, hydroxyapatite (HA), sodium lithium niobate, and boron nitride nanotubes have significant advantages in terms of piezoelectric coefficients, but what deserves the attention of all researchers is that piezoelectric ceramics continue to release various types of ions within biological fluids, and these ions can produce varying degrees of cytotoxicity (Rajabi et al., 2015; Tandon et al., 2018). After doping with 10% BT and 10% HA, an output voltage of 0.8 V can be detected on the hydrogel film (Wu et al., 2023). Moreover, due to the non-degradability and high elastic modulus of piezoelectric ceramic-based tissue engineering scaffolds, most of these materials are doped into various types of polymers or ceramics in the form of piezoelectric particles in order to utilize their excellent piezoelectric properties (Baxter et al., 2009; Bagchi et al., 2014). Electrostatically spun fiber composite scaffolds made of zinc oxide particles and polyurethane lead to improved attachment and proliferation of mouse fibroblasts on the composite scaffolds (Amna et al., 2013). The origin of piezoelectricity in polymers is attributed to the inherent crystalline or chemical structure of the polymer material, which induces a net dipole/charge upon mechanical deformation. Piezoelectric polymers are mainly fabricated in three different forms: membranes, rods or tubes and fibers (Ali et al., 2023). Among them, Polyvinylidene difluoride (PVDF) and its copolymers are characterized by high voltage electrical properties, flexibility, and biocompatibility which makes them good candidates for tissue engineering (Jacob et al., 2018). PVDF and its copolymers have excellent cytocompatibility with positive effects on cell adhesion and proliferation, and can stimulate the differentiation of cells into mature phenotypes, which promotes stem cell-induced tissue repair (Santos et al., 2017). In addition, materials such as poly (l-lactic acid), poly (lactic-co-glycolic acid), polyhydroxybutyrate and polyamides (Nylons, peptides, etc.) have been used as options for the preparation of piezoelectric tissue engineering scaffolds because of their wide availability, slow uptake, non-toxic by-products and excellent degradation properties (Shlapakova et al., 2024). These materials have to be widely used in tissues such as bone, cartilage, and nerve, however, no investigator has yet used them for IVD regeneration. And so far, tissue-engineered scaffolds based on the piezoelectric effect require dynamic loading in order to generate charge (Cai et al., 2021). This is an unavoidable bottleneck in their clinical translation. However, IVDs, which are the main load-bearing structures in the human body, are ideal providers of dynamic loading *in vivo*. This makes the use of piezoelectric materials for the treatment of IDD very attractive.

## 5 Piezoelectric tissue engineering for regenerative IVD repair

Currently, research on the application of piezoelectric materials in tissue engineering is focused on the regeneration of bone, nerve and muscle. A variety of materials have been demonstrated to have high piezoelectric correspondence and can serve as ideal material platforms for demonstrating the concept of electromechanical conversion for tissue engineering. The physiological mechanism of piezoelectric tissue engineering scaffolds to promote tissue regeneration and repair and regulate immunity is very complex, and a variety of physiological processes are involved. Human cells also have a resting potential that is negatively charged internally and positively charged externally under normal conditions. Its dynamic balance is maintained by the transport of sodium and potassium ions. When stimulated by an external electrical pulse, cells can undergo rapid depolarization and repolarization. For these reasons, cell behavior can be controlled by electrical stimulation. Appropriate patterns of electrical stimulation can direct cell growth and regeneration. Stimulation of human mesenchymal stromal cells (hMSCs) by bi-directional currents *in vitro* leads to better osteogenic differentiation and induces the production of vascular endothelial growth factor (VEGF) and BMP-2. Moreover, the effects of selective inhibitors of p38 MAPK or Erk, as well as calcium channel blockers (verapamil and nifedipine), confirmed that MAPK (Erk and p38) and calcium channels are responsible for the osteogenesis promoted by electrical stimulation (Kim et al., 2009).

However, no investigator has yet reported piezoelectric tissue engineering for repair of degenerated IVD. Nevertheless, most researchers have verified the feasibility of electrical stimulation to promote IVD repair through various *in vitro* experiments. This provides a theoretical basis for implanting piezoelectric materials with self-powered properties for regenerative repair of IVD. For IDD, disruption of ECM synthesis and metabolic balance as well as dysregulation of inflammatory mediators are the main reasons for its development. Implanted piezoelectric tissue-engineered scaffolds can help restore cells within the IVD by providing continuous electrical stimulation. An *in vitro* study assessing the effect of electrical stimulation on the production of IVD matrix macromolecules through human NP cells found that electrical stimulation upregulated the production of IVD-matrix macromolecules aggregated glycans, type II collagen. and sGAG through the mechanism of BMPs, and that electrical stimulation synergized with BMP-7 to increase the upregulation of these ECM macromolecules of the IVD (Wang et al., 2017). Similarly electrically stimulated isolated porcine IVD were able to exhibit extensive annular regeneration and prevented NP protrusion. Gene expression showed an increase in ECM markers and the anti-inflammatory cytokine interleukin-4 (IL-4) and a decrease in pro-inflammatory markers and pain markers in electrically stimulated IVD (Kanan et al., 2022). On the other hand, IL-1β-mediated positive effects in terms of morphological phenotypes and kinetic properties after the resultant electrical stimulation of human NP cells (Kim et al., 2021). Selective electrical stimulation of a degenerative inflamed human fibrocyclic cell model confirmed that the stimulated cells inhibited the secretion of inflammatory cytokines and downregulated the activities of ECM-modifying enzymes and matrix metalloproteinase-1 (MMP-1) (Shin et al., 2019). IL-1β is a major regulator secreted by macrophages, mediates the inflammatory response of the NP, and is that it is a major regulator of IVD degeneration. However, exposure of IL-1β-stimulated AF cells to electrical stimulation at 500 mV/mm inhibited the production of MMP-1, IL-6, VEGF and TIMP-1 (Kim et al., 2013). Similarly, macrophage-mediated production of IL-6 and IL-8 in the disc was significantly inhibited in a macrophage-mediated model of NP inflammation after electrical stimulation (Kim et al., 2022). In addition, cells within the IVD undergoing electrical stimulation also attenuated the IL-1α-induced upregulation of genes expressed in the degenerative IVD. expression of IL-1α-related genes of IL-6 in NP cells was significantly reduced in AF cells by MMP13, and concomitantly reduced IL-1α-induced gene expression of IL-17A and MMP2 in NP cells and NFκB in AF cells (Miller et al., 2016). The MAPK and NF-kB signaling pathways play important roles in the development of IDD, and electrical stimulation can control the expression of each factor by controlling MAPK and NF-kB. It can effectively regenerate NP cells by decreasing their inflammatory expression during IVD degeneration, which ultimately reduces the stimulation of intervertebral nerves and decreases the expression of pain-related receptors and factors (Rubin et al., 2006; Kaur et al., 2011).

**FIGURE 2:**
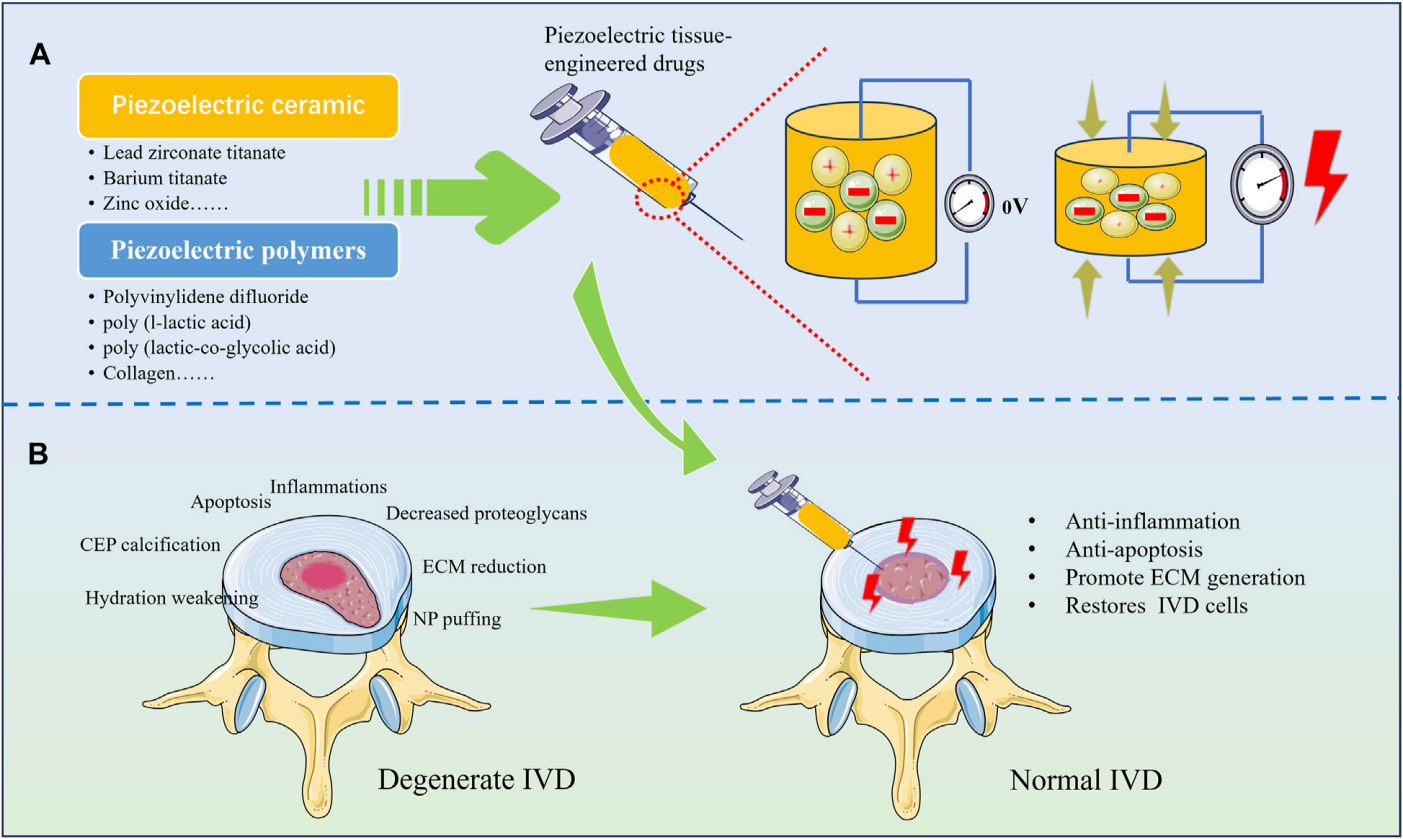
Various types of piezoelectric materials for repairing degenerated IVD. **(A)** Tissue-engineered drugs prepared from piezoelectric materials have the ability to generate electricity under pressure; **(B)** Regenerative repair can be achieved by implanting piezoelectric materials into degenerated IVD.

These results confirm the feasibility of piezoelectric tissue engineering for the treatment of IDD, which currently relies mostly on extracorporeal electrodes for electrical stimulation of the IVD. However, for patients with IDD, most of the extracorporeal electrodes are implanted in the body by means of needling, which makes it difficult for electrical stimulation to accurately act within the IVD, and needling is difficult to provide stable electrical stimulation for a long period of time. The risk of infection associated with needling is also not negligible. However, by implanting a tissue-engineered scaffold made of piezoelectric material with self-powered properties into the IVD, electrical stimulation can be generated by axial pressure from the spine, which can help to restore the number of cells in the IVD, regulate the immune microenvironment, and alleviate the pain to achieve the purpose of delaying or even reversing the IDD (Figure 2). Stem cell-based biotherapies are also seen as a promising approach in the treatment of IDD and have been extensively studied. By combining piezoelectric materials with stem cells, electrical stimulation can be used to further enhance the regenerative repair capacity of stem cells. For example, researchers have found that electrical stimulation obtained by converting ultrasound energy via piezoelectric nanoparticles barium titanate can induce stem cell differentiation via calcium ion channels and subsequent signalling (Liu et al., 2020). Although tissue engineering with piezoelectric materials has now been shown to promote growth, differentiation and proliferation, what still deserves the attention of researchers is the safe range of electrical stimulation. Higher electrical stimulation can adversely affect cell growth and even lead to death (Chen et al., 2019). Therefore, in designing piezoelectric tissue engineering scaffolds for IDD treatment, further consideration still needs to be given to the material’s ability to generate electricity, the magnitude and duration of external mechanical stress, and the site of action. This still needs to be further explored to achieve the desired tissue regeneration.

## 6 Conclusion

Currently, IDD caused by a variety of etiologic factors seriously affects people’s health and increases the burden on society. Although regenerative therapies, such as stem cell therapy and exosome therapy, have shown better promise in delaying or reversing IDD than traditional drug and surgical treatments, they still have many shortcomings. In recent years, researchers have begun to focus on various mechanotransduction mechanisms within the IVD and have found that SGP plays an important role in maintaining the normal structure and function of the IVD. Thus, electrical stimulation therapy is considered a feasible new option for the treatment of IDD. Moreover, piezoelectric biomaterials with good biocompatibility and autogenous properties can promote cell proliferation and differentiation and regulate immune response through calcium channels and MAPK and other related mechanisms, and have been widely used in the regenerative repair of various tissues, such as bone, cartilage and nerve. Moreover, a series of *in vitro* experiments have confirmed that electrical stimulation can the regeneration of NP cells, upregulate the expression of ECM-related molecules, inhibit the secretion of inflammatory factors, and decrease the expression of pain-related receptors and factors. Thus the use of piezoelectric tissue engineering techniques for IVD repair has some research implications. We hypothesized that the ability of tissue-engineered scaffolds of piezoelectric materials implanted into IVD to intrinsically delay or reverse IDD may be superior to direct electrical stimulation *in vitro*.

## Data Availability

The original contributions presented in the study are included in the article/Supplementary material, further inquiries can be directed to the corresponding author.
